# Electron energy constancy verification using a double‐wedge phantom

**DOI:** 10.1120/jacmp.v4i3.2516

**Published:** 2003-06-01

**Authors:** Derek M. Wells, Philip J. Picco, Will Ansbacher

**Affiliations:** ^1^ Department of Medical Physics British Columbia Cancer Agency–Vancouver Island Centre 2410 Lee Avenue Victoria British Columbia Canada V8R 6V5

**Keywords:** electron energy constancy, diode array

## Abstract

Routine constancy checks of electron energy are often time consuming because of the necessity to measure a dose at two depths. A technique is described that uses a double‐wedge shaped phantom positioned on a Profiler™ diode array for measuring an electron energy constancy metric similar to $R_{50}$. The double‐wedge electron profiles are invariant to phantom alignment in the wedge direction, unlike single wedge techniques, and the sensitivity of the technique is similar to water‐based depth‐dose measurements over an energy range of 6 to 20 MeV. Reproducibility results ranging from 0.01 to 0.03 cm were achieved for measurements taken over the course of 1.5 yrs. The technique is efficient in that only one phantom setup is required for all electron energies.

PACS number(s): 87.53.–j, 87.66.–a

AAPM Task Group 40^1^ recommends that each teletherapy electron beam be evaluated monthly to ensure that its penetrative ability remains consistent with commissioning data. A routine test for determining an energy constancy metric similar to the depth at 50% maximum output, $R_{50}$, becomes tedious for multiple electron energy machines if solid water slabs are utilized. That is, different thicknesses of attenuation material are required to characterize each electron energy. Various approaches have been undertaken to streamline the measurement process. Moyer^2^ introduced a technique for producing an electron depth‐ionization curve that involved an aluminum wedge placed on film. The electron energy was correlated with the shape of the curve on film. As with any film‐based technique, the reproducibility of the resultant metric is influenced by variations in film processing conditions. Furthermore, separate films must be exposed at each energy, which is time consuming. Filmless approaches using a polystyrene wedge‐shaped phantom placed upstream of an ion chamber array^3,^, ^4^ (Thebes™ Model 7000, Victoreen, Inc., Cleveland, OH) and a diode array^5^ (Profiler™ Model 1170, Sun Nuclear Corporation, Melbourne, FL) have been introduced. Techniques involving a wedge placed upstream of a 9.5 cm diameter parallel plate chamber^6^ as well as a 10 cm long diagnostic CT ion chamber^7^ have also been used to acquire an energy constancy metric. With these techniques, the constancy metric is expressed as a ratio of electrometer reading with and without the upstream wedge. More elaborate electron detector devices have been used to measure depth dose curves including a commercially available energy monitor (Geske 3405, PTW, Freiburg, Germany) consisting of nine parallel plate detectors that has been evaluated by several groups^8,^, ^9^ and a detector device consisting of 12 scintillating fibers embedded into an acrylic phantom.^10^

In this paper, a technique for measuring an electron energy constancy metric similar to $R_{50}$ using a double‐wedge shaped phantom positioned on a Profiler™ diode array is described. The double‐wedge electron profiles are invariant to phantom alignment in the wedge direction, unlike single wedge techniques, and the sensitivity of the technique is similar to water‐based methods over an energy range of 6 to 20 MeV.

The phantom (Fig. 1) consists of two acrylic wedges mounted to an acrylic base and separated by an air gap of 1.5 cm in the wedge direction. The overall width of the phantom in the nonwedge direction is 25 cm to allow for full side scatter conditions to the diode array. A wedge angle of 45° provides measurement sensitivity similar to that of water‐based measurements; a 30° angle at the phantom base increases the measurement sensitivity for low electron energy beams (4 to 6 MeV). Two pins were added to the bottom of the phantom base to fit into the build‐up alignment holes of a Profiler™ diode array. The array consists of 46 solid state, radiation‐hardened diodes spaced 0.5 cm apart and has an inherent build‐up to the detector junction of $0.9\pm 0.1\;g/{\text{cm}}^{2}$. Some of the performance characteristics of this diode array for photons have been evaluated by Zhu *et al.* ^11^

**Figure 1:**
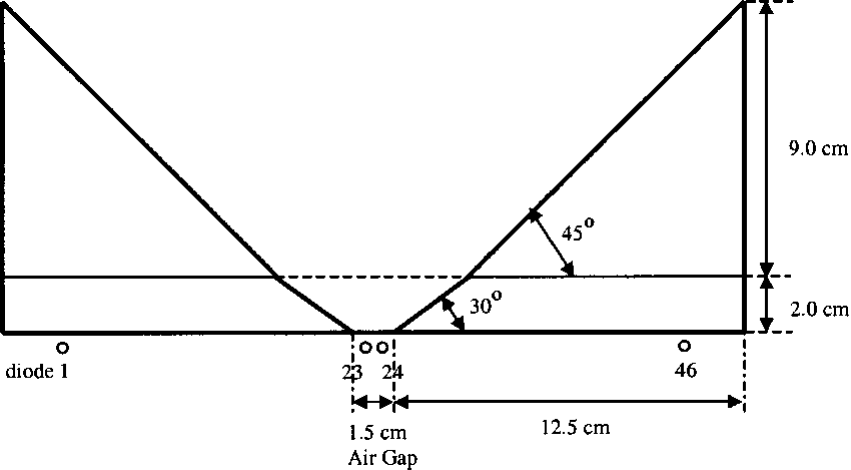
Double‐wedge design.

All measurements were taken at a distance of 110 cm from the source to the diode array surface, with a 20×20 cm electron applicator and standard insert. The center of the diode array (a point halfway between diodes 23 and 24) was aligned with the cross‐hair projection of the linear accelerator. The double‐wedge phantom was positioned over the diode array such that the 1.5 cm air gap overlaid the two center diodes.

Due to the varying sensitivity of individual diodes, a relative calibration factor must be determined for each of the 46 diodes. This is a standard operating procedure for the Profiler™ in which each diode is referenced to diode 1. Under broad‐beam conditions (25×25 cm applicator), diode outputs ranged from 0.85 to 1.35. Initially, separate calibration factors were determined for all nominal electron energies: 6, 9, 12, 16, and 20 MeV; however, little energy dependency was exhibited by the diodes. A single calibration factor (averaged over all energies) was then calculated for each diode, with a corresponding standard deviation of only 0.2% [Fig. 2(a)]. The long‐term stability of the diode sensitivities was determined by recalculating the calibration factors at 6‐month intervals over a one‐year period. The standard deviation of the calibration factor differences over a six‐month period was 0.3% and over a 12‐month period was 0.8% [Fig. 2(b)]. Our policy was to establish new calibration factors for the diodes every six months. If the above errors associated with the calibration factors are added in quadrature, then an error in the energy constancy metric of about 0.01 cm is introduced, which was considered acceptable.

**Figure 2:**
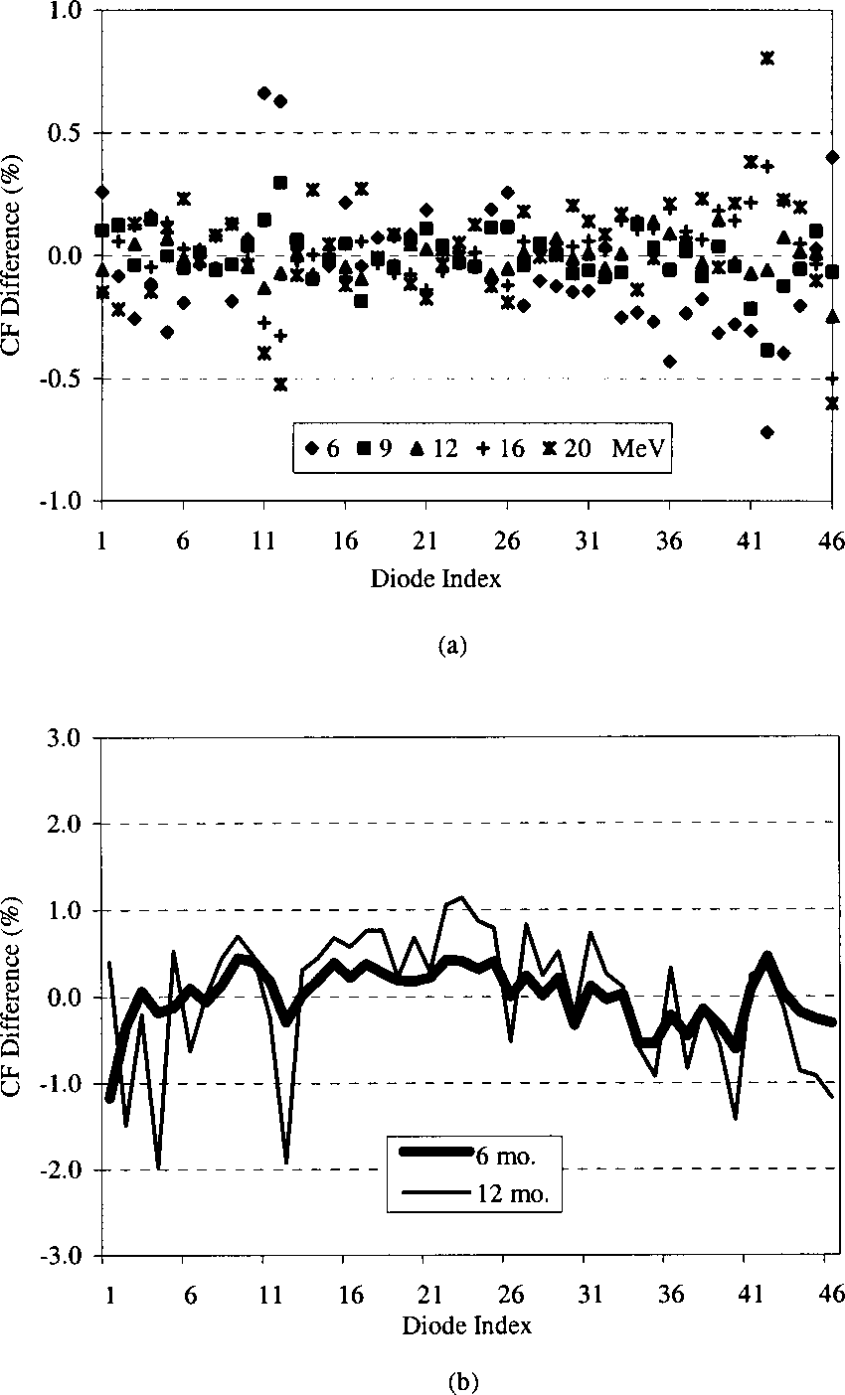
(a) Comparison of mean and individual calibration factors (CF) for all nominal electron energies. (b) Change in mean calibration factors over a period of 1 yr.

The double‐wedge electron profiles were transferred to a spreadsheet program for analysis. Each profile was normalized to the average diode output in the air gap region of the double‐wedge phantom (Fig. 3). The full width of the electron profile at the 50% relative output level was determined by linear interpolation, and one half of this value defined the energy constancy metric, ${\text{EC}}_{50}$.

**Figure 3:**
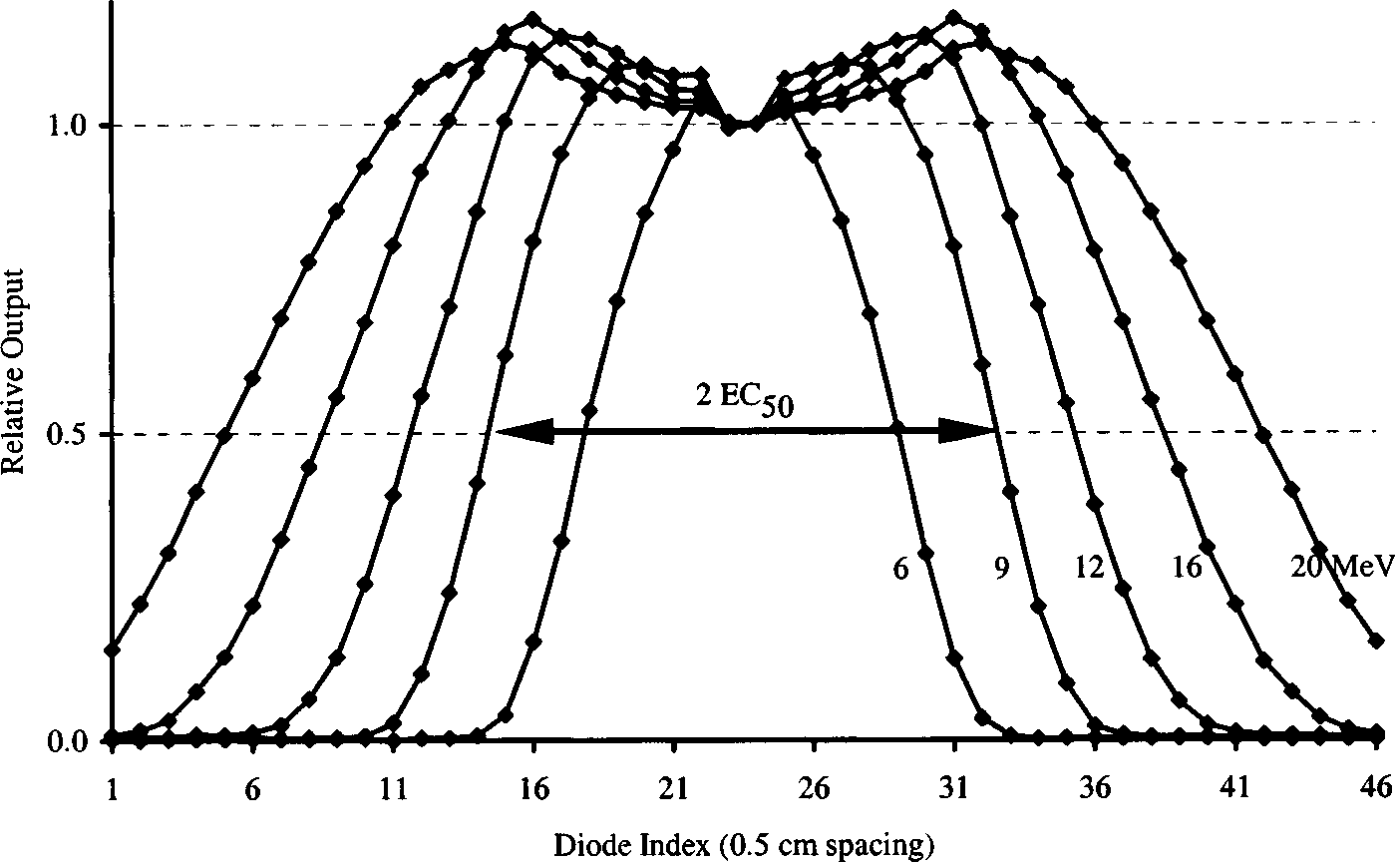
Double‐wedge electron profiles measured with diode array. Profiles are normalized to 1.0 in the region corresponding to the air‐gap, not the maximum output. The width of the profile at the 50% level (as denoted by the arrow for 9 MeV) was determined by linear interpolation. The energy constancy metric, ${\text{EC}}_{50}$, was defined as one half of this value.

This approach to determining an energy constancy metric was not dependent on the exact placement of the wedge phantom on the diode array. Tests were performed with the double‐wedge phantom offset from central axis by ±0.25 cm in the wedge direction with no significant change in the calculated value of the energy metric (Table I). If the profiles were normalized to the maximum output at each energy, as is the case with single wedge techniques, then the metric would potentially be sensitive to wedge position. In particular, there is no guarantee that a diode would be situated exactly at the maximum output for low electron energies with narrow output peaks.

**Table I:** Dependency of EC50 on double‐wedge phantom position.

Nominal energy (MeV)	${\text{EC}}_{50}$ (cm)	${\text{EC}}_{50}$(–)^a^ (cm)	${\text{EC}}_{50}$(+)^a^ (cm)
6	2.79	2.80	2.80
9	4.53	4.53	4.53
12	5.91	5.91	5.91
16	7.49	7.49	7.48
20	9.23	9.22	9.22

^a^ (±) indicates a ±0.25 cm shift of the phantom in the wedge direction.

The flatness and symmetry of each open electron beam profile was assessed using the same Profiler™ setup as the double‐wedge phantom measurements. It was decided that because the ${\text{EC}}_{50}$ metric is only a constancy measure and not intended to be equal to $R_{50}$, no correction was required for the double‐wedge electron profiles for off‐axis differences in open beam output.

The sensitivity of the double‐wedge phantom technique for measuring energy constancy is equal to or better than water‐based measurements (Fig. 4), where the mean ${\text{EC}}_{50}$ value is plotted against the mean $R_{50}$ value obtained during commissioning. That is, the slope of ${\text{EC}}_{50}$ versus $R_{50}\geq 1$. The sensitivity realized with the double‐wedge phantom is dictated by the wedge angle, with smaller angles increasing the sensitivity. However, the double‐wedge design is constrained by the total length of the linear diode array (22.5 cm). Increased sensitivity could be achieved with this diode array by using a smaller angled double‐wedge phantom for low electron energies; however, this would defeat the advantage of having a universal tool.

**Figure 4:**
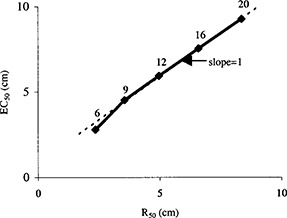
Sensitivity of double‐wedge technique vs water‐based technique for electron energies ranging from 6 to 20 MeV.

Electron energy constancy data were obtained on a monthly basis for a period of 1.5 yrs on four linear accelerators (Clinac 21EX Millenium Series, Varian Medical Systems, Palo Alto, CA). Reproducibility results are summarized in Table II, with standard deviations ranging from 0.01 to 0.03 cm. The four linear accelerators were “energy matched” during acceptance testing to better than ±0.1 cm at four depths corresponding to 90%, 80%, 50%, and 30% relative output. The standard deviation of the measured $R_{50}$ values ranged from 0.02 to 0.04 cm; hence, it was feasible to use a mean ${\text{EC}}_{50}$ value for each electron energy. Reproducibility results using the mean values for ${\text{EC}}_{50}$ yielded standard deviations ranging from 0.01 to 0.05 cm. Therefore, the 99% confidence limits (3 standard deviations) for the estimate of ${\text{EC}}_{50}$ at each electron energy was still below the 2 mm tolerance level specified in the TG 40 recommendations.^1^ The conversion from ${\text{EC}}_{50}$ to $R_{50}$ is shown in Fig. 4. The 0.5 cm diode spacing and the acrylic double‐wedge angles are adequate to determine this metric reproducibly. Diode spacing greater than 0.5 cm and/or a larger wedge angle would potentially result in a failure of the linear interpolation algorithm to accurately estimate ${\text{EC}}_{50}$ at low electron energies.

**Table II:** Reproducibility of energy constancy metric, ${\text{EC}}_{50}$, for four linear accelerators. Measurements were taken on a monthly basis for a period of 1.5 yrs. Only machines 1 and 2 had the capability of producing 20 MeV electron beams.

Nominal energy (MeV)	$R_{50}$ ^a^ (cm)	${\text{EC}}_{50}$ ^a^ (cm)	$S.D._{1}$ (cm)	$S.D._{2}$ (cm)	$S.D._{3}$ (cm)	$S.D._{4}$ (cm)
6	2.35±0.02	2.80±0.02	0.01	0.01	0.01	0.01
9	3.55±0.04	4.52±0.04	0.01	0.01	0.01	0.01
12	4.97±0.03	5.94±0.02	0.02	0.02	0.02	0.02
16	6.59±0.04	7.54±0.05	0.02	0.03	0.03	0.02
20	8.36±0.03	9.26±0.01	0.01	0.01	—	—

^a^ Average $R_{50}$ and ${\text{EC}}_{50}$ values and respective standard deviations are shown.

The Profiler™ is used for other monthly quality assurance procedures, including flatness and symmetry measurements for both electron and photon beams; hence, the preparation time required for the double‐wedge measurements is minimal. Finally, the technique only requires that the user enter the treatment vault once thus providing efficient time usage for this routine quality assurance task.
